# Ivermectin Inhibits Bovine Herpesvirus 1 DNA Polymerase Nuclear Import and Interferes With Viral Replication

**DOI:** 10.3390/microorganisms8030409

**Published:** 2020-03-13

**Authors:** Sohail Raza, Farzana Shahin, Wenjun Zhai, Hanxiong Li, Gualtiero Alvisi, Kui Yang, Xi Chen, Yingyu Chen, Jianguo Chen, Changmin Hu, Huanchun Chen, Aizhen Guo

**Affiliations:** 1 The State Key Laboratory of Agricultural Microbiology, Huazhong Agricultural University, Wuhan 430070, China; sohail.raza@uvas.edu.pk (S.R.); f.shaheen64@yahoo.com (F.S.); wjzhai94@gmail.com (W.Z.); lhx924@hotmail.com (H.L.); chenxi@mail.hzau.edu.cn (X.C.); chenyingyu@mail.hzau.du.cn (Y.C.); chenhch@mail.hzau.edu.cn (H.C.); 2 Department of Microbiology, University of Veterinary and Animal Sciences, Lahore 54000, Pakistan; 3 College of Veterinary Medicine, Huazhong Agricultural University, Wuhan 430070, China; chenjg@mail.hzau.edu.cn (J.C.); hcm@mail.hzau.edu.cn (C.H.); 4 Department of Molecular Medicine, University of Padua, 35121 Padua, Italy; gualtiero.alvisi@unipd.it; 5 Department of Pathobiological Sciences, School of Veterinary Medicine, Louisiana State University, Baton Rouge, LA 70803, USA; 6 Key Laboratory of Development of Veterinary Diagnostic Products, Key Laboratory of Ruminant Bio-Products of Ministry of Agriculture and Rural Affairs, Wuhan 430070, China; 7 Hubei International Scientific and Technological Cooperation Base of Veterinary Epidemiology, International Research Center for Animal Disease, Ministry of Science and Technology of the People’s Republic of China, Wuhan 430070, China

**Keywords:** BoHV-1, nucleocytoplasmic shuttling, Ivermectin, antiviral, NLS, DNA polymerase holoenzyme, pUL30, pUL42

## Abstract

*Bovine herpesvirus1* (BoHV-1) is a major bovine pathogen. Despite several vaccines being available to prevent viral infection, outbreaks are frequent and cause important economic consequences worldwide. The development of new antiviral drugs is therefore highly desirable. In this context, viral genome replication represents a potential target for therapeutic intervention. BoHV-1 genome is a dsDNA molecule whose replication takes place in the nuclei of infected cells and is mediated by a viral encoded DNA polymerase holoenzyme. Here, we studied the physical interaction and subcellular localization of BoHV-1 DNA polymerase subunits in cells for the first time. By means of co-immunoprecipitation and confocal laser scanning microscopy (CLSM) experiments, we could show that the processivity factor of the DNA polymerase pUL42 is capable of being autonomously transported into the nucleus, whereas the catalytic subunit pUL30 is not. Accordingly, a putative classic NLS (cNLS) was identified on pUL42 but not on pUL30. Importantly, both proteins could interact in the absence of other viral proteins and their co-expression resulted in accumulation of UL30 to the cell nucleus. Treatment of cells with Ivermectin, an anti-parasitic drug which has been recently identified as an inhibitor of importin α/β-dependent nuclear transport, reduced UL42 nuclear import and specifically reduced BoHV-1 replication in a dose-dependent manner, while virus attachment and entry into cells were not affected. Therefore, this study provides a new option of antiviral therapy for BoHV-1 infection with Ivermectin.

## 1. Introduction

*Bovine herpesvirus 1* (BoHV-1) belongs to the family *Herpesviridae* and subfamily *Alphaherpesvirinae*. The members of this family are large, enveloped and double-stranded DNA viruses. Among them, the BoHV-1 is an important member that causes severe economic losses to the cattle industry around the world [1]. BoHV-1 infection leads to lesions in the respiratory, nervous, reproductive, and digestive systems in infected-bovines [2]. During upper respiratory tract infection, this virus causes acute rhinotracheitis, conjunctivitis, nasal discharge, dyspnea, and mucosal damage. Most importantly, BoHV-1 infection paves the way for secondary bacterial infections of the respiratory tract. This life-threatening polymicrobial respiratory disease is referred to as bovine respiratory disease complex (BRDC) which causes heavy economic losses to the bovine industry throughout the world [3,4]. Moreover, BoHV-1 can establish lifelong latency in the trigeminal ganglion (TG) of the infected animals. Stress condition in animals due to shipping, weaning or weather change may reactivate BoHV-1 from TG [5,6] and cause shedding of viruses through ocular and nasal secretions that become an important source of viral spreading to susceptible animals. No drugs are available to directly target BoHV-1 infection and, despite the availability of several types of vaccines to prevent BoHV-1 infection, outbreaks are still widespread because of the suboptimal protection against infection conferred by vaccination [7]. Therefore, there is a growing need to develop novel drugs that can be used to treat BoHV-1 infected animals.

Viral genome replication is one of the most common targets for antiviral therapy [8,9]. The molecular details of BoHV-1 gene expression and replication are still largely unknown, but it can be hypothesized that, as well described for other *Herpesviridae* members, herpesviruses genome replicates in the nucleus of infected cells with the help of a number of cellular and viral proteins. Most of such viral proteins are conserved in all herpesviruses and are believed to function similarly [10]. The latter includes an origin binding protein, a DNA polymerase holoenzyme composed of a DNA-dependent DNA polymerase catalytic subunit and a DNA polymerase accessory protein (PAP) conferring processivity to the holoenzyme, as well as a trimeric helicase/primase complex and a single-stranded (ss) DNA-binding protein. The above-mentioned proteins have been identified for several human *Herpesviridae* family members by means of *ori*Lyt-dependentreplication trans complementation assays [11]. Importantly, the proteins involved in the herpesvirus DNA replication must be transported into the nuclei after their synthesis in the cytoplasm in order to participate in viral DNA synthesis [10]. In eukaryotic cells, the nucleus is separated from the cytoplasm by a double membrane nuclear envelope, the passage where large multiprotein channels named nuclear pore complexes (NPCs) exist. Through these NPCs, translocation of ions and small proteins can occur via passive diffusion, whereas translocation of larger protein complexes (above 60kDa) is a signal and energy-dependent process. In the latter scenario, cellular transporters, belonging to the importin superfamily, are active transport molecules which were found across the nuclear pore complexes (NPCs) [12] and can recognize specific signals located on the cargoes to be either imported (nuclear localization signals—NLSs) or exported (nuclear export signal—NESs) before docking and translocation trough the NPC [13]. Several different importins can recognize cargoes bearing specific NLSs, but the best-characterized and most widely used pathway relies on the importin α/β heterodimer, whereby importin α recognizes basic “classical” NLSs (cNLSs), resembling the one originally described on Simian Virus 40 Large Tumor Antigen (PKKKRKV-132) [14], and mediates translocation thorough the NPC [15]. Previous studies have characterized the nuclear import of several *Herpesviriade* members DNA polymerases and showed that in all cases nuclear transport is mediated by importin α/β via the recognition of cNLSs located on the polymerase subunits [10]. However, while for certain viruses, including Human Cytomegalovirus (HCMV) and Human Herpes Simplex type 1 (HSV-1), both the DNA polymerase catalytic subunit and its PAP bear functional cNLSs can be independently translocated into the nucleus [16,17,18,19]; in other cases, such as in Epstein–Barr virus (EBV), Kaposi’s sarcoma-associated herpesvirus (KSHV) and Pseudorabies virus (PRV), the catalytic subunit lacks a functional NLS and can only be transported into the nucleus by the PAP in the context of a pre-assembled DNA polymerase holoenzyme [20,21,22]. To date, little is known regarding the biochemical properties of the BoHV-1 DNA polymerase pUL30 and its PAP pUL42, and the molecular determinants of their nuclear transport during viral infection.

Ivermectin (IVM) is a broad-spectrum antiparasitic drug used in both humans and animals, which has been recently shown to inhibit the importin α/β nuclear transport pathway by mediating the dissociation of importin αfrom importin βIVM, which exhibited important antiviral activity against human immune virus 1 (HIV-1) and dengue virus, by affecting nuclear transport of HIV-1 intergrase and dengue virus (DENV) nonstructural protein 5 (NS5). IVM also affected DENV-2 virus replication in *Aedes albopictus*. In addition, IVM exhibited a broad spectrum antiviral activity against several members of the Flavivirus genus, including Japanese encephalitis virus, West Nile virus (WNV), tick-borne encephalitis virus, Chikungunya virus, and Zika virus [23]. Given the well-established role of importin α/β in herpesviral DNA polymerase nuclear import, we hypothesized the possibility to use IVM in order to interfere with the nuclear import of BoHV-1 DNA polymerase, thus blocking viral replication. To test such a hypothesis, we transiently expressed both BoHV-1 DNA polymerase pUL30 and its PAP pUL42 in mammalian cells for the first time. Our results showed that the two proteins can physically interact in the absence of any other viral protein. A bioinformatics analysis revealed that pUL42, but not pUL30, possessed a putative cNLS at the C-terminus, and was localized in the cell nucleus when individually expressed, while simultaneous expression of both proteins resulted in co-localization within the cell nucleus. Importantly, treatment with IVM functionally impaired nuclear import of pUL42 and strongly reduced BoHV-1 replication in a dose-dependent fashion, without affecting viral attachment and entry. 

## 2. Materials and Methods 

### 2.1. Plasmids

For the expression of recombinant proteins in eukaryotic cells, the full-length UL30 (GenBank accession no: CAA06103.1) and UL42 (GenBank accession no: CAA06091.1) genes were amplified from the DNA template extracted from BoHV-1 strain IBRV HB06 (isolated by this lab and stored as no. CCTCC V201024 in the Tissue Culture Collection Center of China at Wuhan University) using primer sets:

UL30HA(F): 5′GCAAGCTTGCCACCATGTACCCATACGATGTTCCAGATTACGCTGACAGAGACTGCGAACAGGGC’3;
UL30HA(R): 5′CGGAATTCTCAACTTTGATGGGGAGCTGCTGCTAGAATACCGAAGGCTGT’3*;* UL42FLAG(F): 5′GCAAGCTTGCCACCATGGATTACAAGGATGACGACGATAAGCTGCAGCCCCCCTCGCAT’3;
UL42FLAG(R):5′CGGGATCCTTAAGGAGTTTCGCCCCCCTCCCCG’3.

The primers contained the molecular tags HA and FLAGat the N-terminal of UL30 and UL42 proteins, respectively. The amplified genes were cloned into the eukaryotic expression plasmid pcDNA4 (Thermo Fisher, Rockford, IL, USA) and resulting recombinant expression plasmids were named as pcDNA4-UL30-HA and pcDNA4-UL42-FLAG, respectively. All constructs were confirmed by PCR and DNA sequencing of PCR products.

### 2.2. Transfection of Cells with UL30-HA and UL42-FLAG Expressing Plasmids

Madin–Darby Bovine Kidney (MDBK) cells were seeded onto 6-well plates (Corning, NY, USA) in DMEM medium (Hyclone, PA, USA) supplemented with 10% FBS (Gibco, MD, USA) at a density of 2 ×10^5^ cells per well overnight before transfection and grown to 50% confluence by the following day. Cells were transfected separately or co-transfected with recombinant plasmids expressing UL30-HA and UL42-FLAG. Transfection was carried out using Lipofectamine™ 2000 Transfection Reagent (Invitrogen, Carlsbad, CA, United States) according to the manufacturer’s protocol. After 6 h, fresh RPMI-1640 medium containing 10% FBS was added. At 24 h post transfection, cells were processed for immunofluorescence and Western blot analysis as described below.

### 2.3. Co-Immunoprecipitation Assay and Western Blot Analysis 

MDBK cells grown in 6-well tissue culture plates were either mock-transfected or transfected withplasmids encoding for UL30-HA and UL42-FLAG. At 24 h post transfection, cells were harvested in 50 µl of lysis buffer (1% Triton X-100, 1 mM ethylenediaminetetraacetic acid (EDTA), 50 mM Tris, and 150 mM NaCl) containing 1× protease inhibitor cocktail (Sigma-Aldrich, Shanghai, China). Cell lysates were incubated for 7 min on ice and clarified by centrifugation at 160× g for 10 min at 4°C. Co-immunoprecipitation (Co-IP) and Western blot analysis were performed as described previously [1,24]. Briefly, the Co-IP was performed using anti-HA/FLAG monoclonal antibody-conjugated magnetic agarose beads (MBL, 287 Nagoya, Japan), and the proteins were separated on 10% sodium dodecyl sulfate-polyacrylamide gel electrophoresis (SDS-PAGE) before being electro-blotted onto polyvinylidene fluoride (PVDF) membranes (Millipore, Billerica, MA, USA). The membranes were blocked in 5% skim milk and probed with rabbit anti-FLAG (1:1000; Beyotime, Haimen, China) and mouse anti-HA antibody (1:1000; Beyotime, Haimen, China) or antibody to β-actin as an internal reference (1:1000; Beyotime, Haimen, China). Membranes were further incubated with goat anti-rabbit and goat anti-mouse secondary antibodies conjugated with horseradish peroxidase (HRP; 1:5000; Southern Biotech, Birmingham, MI, USA). Proteins were visualized using enhanced chemiluminescent substrate (Thermo Fisher Scientific, Rockford, IL, USA) according to the manufacturer’s instruction.

### 2.4. Immunofluorescence and Confocal Laser Scanning Microscopy (CLSM)

MDBK cells grown on glass coverslips were transfected as described above with the expression plasmids encoding the UL30-HA and UL42- FLAG proteins with or without the drug IVM. At 24 h post transfection, cells were fixed in 4% paraformaldehyde in phosphate-buffered saline (136.9 mM NaCl, 2.7 mM KCl, 7.0 mM Na_3_PO4, 0.9 mM Na_3_PO_4_ at pH 7.4) at 37°C for 20 min and permeabilized with 0.2% Triton X-100 in PBS for 10 min. Cells were subsequently incubated in blocking buffer (1% bovine serum albumin (BSA) (Biosharp, Hefei, China) and 0.1% Tween 20 in PBS) for 2 h. Cells were subsequently incubated with the primary antibodies including mouse anti-HA and rabbit anti-FLAG (Beyotime, Haimen, China) at 1:500 dilution in blocking buffer. Finally, cells were stained with secondary antibodies, including Cy3-conjugated goat anti-mouse and fluorescein isothiocyanate (FITC)-conjugated goat anti-rabbit (Beyotime, Haimen, China) at 1:1000 dilutions in blocking buffer. The DNA was counterstained with 4, 6-diamidino-2-phenylindole (DAPI). After each step, cells were washed three times with 1× PBS. The fluorescent signals were observed using Zeiss LSM 880 CLSM (Carl Zeiss, Jena, Germany), equipped with a 63x objective. Images were processed using Image J (NIH) as described previously [25].

### 2.5. Identification of cNLSs with Bioinformatics Analysis

The primary sequences of BoHV-1 pUL30 and pUL42 were scanned using software cNLS mapper [26] to identify putative cNLSs as described in [27].

### 2.6. Cells and Viruses

MDBK cells were grown and maintained in complete Dulbecco’s Modified Eagle Medium (DMEM) supplemented with 10% fetal bovine serum, 100 IU/mLpenicillin and 100 µg/mLstreptomycin at 37°C in 5% CO_2_. The IBRV HB06 strain was propagated and maintained as described previously [28].

### 2.7. Compounds

Ivermectin (IVM, 22, 23-dihydroavermectin B_1a_; Abcam, Cambridge, UK) stock solution was prepared in dimethyl sulfoxide (DMSO) at 50 mM and stored in aliquots at −20°C for up to 1month. Before use, the IVMsolution was equilibrated to room temperature.

### 2.8. MTT Cell Proliferation and Cytotoxicity Assay

IVM cytotoxicity was determined by a cell proliferation assay using an MTT cell proliferation and cytotoxicity test kit (Beyotime, Hainan, China) according to the manufacturer’s instruction. Briefly, MDBK cells were seeded on a 96-well cell culture plate; after 24 h, the cells were treated with increasing concentrations of IVM (0–25 µM) or control in triplicate wells and incubated at 37°C with5% CO_2_ for 48 hrs. MTT solution was added and cells were further incubated for 4 h before reading the absorbance at 570 nm with a micro plate reader. The percentage of viable cells was calculated as described elsewhere [29]. Untreated cells were considered as 100% viable.

### 2.9. Antiviral Activity of IVM in Cell Culture

MDBK cells were seeded on cell culture flasks. After 20–24 h, the cells were washed with phosphate buffered saline (PBS) and infected with BoHV-1 at a multiplicity of infection (MOI) of 0.1 or 1 PFU per cell along with increasing concentrations of IVM. At 2 h post infection (PI), the cells were washed three times with PBS to remove unattached viruses. After washing, the maintenance medium was added along with the various concentrations of IVM mentioned above. At different time points, cell-associated viral progeny was collected by three cycles of freeze/thawing, mixed with cell culture supernatant, and viral titers were quantified by plaque reduction assays (PRAs).

### 2.10. Plaque Reduction Assays (PRAs)

PRAs were performed in a 6-well cell culture plate. MDBK cells were cultured up to a confluency of 90%, washed three times with PBS; the samples at 10-fold serial dilution were added and incubated at 37°C and 5% CO_2_ for 2 hrs. After incubation, the inocula were removed and cells were washed with PBS three times. Afterward, the cells were overlaid with agar medium and incubated for 48 h; then the cells were fixed with 10% neutralized formalin and plaques were counted after staining with crystal violet. 

### 2.11. Effect of IVM on BoHV-1 Binding

Binding assays of BoHV-1 virions to the cell membrane in the presence of IVM were performed at 4°C. The BoHV-1 virions were pre-incubated with various concentrations of IVM at 37°C for 1 h, and they were subsequently added to MDBK cells at MOI of 0.1 PFU per cell. These cells were incubated at 4°C for an additional 1 h, and the unbound viruses were removed by washing with PBS, followed with the addition of cell maintenance media without IVM and further incubated at 37°C for 48 h. The progeny viruses were quantified with PRAs according to the above-mentioned method.

### 2.12. Effect of IVM on Virus Penetration

MDBK cells were incubated at 4°C for 1 h with BoHV-1 at MOI 0.1 PFU per cell to allow the viruses to attach to the cell membrane. The unbound viruses were removed and the cells were treated with various concentrations of IVM at 37°C and 5% CO_2_ for 30 min. The residual viruses were washed out with low pH citrate buffer (pH 3.0) [30], followed with the addition of cell maintenance media without IVM, and cells were further incubated at 37°C for 48 hrs. The production of progeny virus was determined by PRAs as described above.

### 2.13. Statistical Analysis

All cellular culture-based experiments were independently repeated at least three times. The data are presented as mean ± standard deviation (SD). The statistical significance values are defined as * *p* <0.05, ** *p* <0.01, ***p <0.001. The statistical significance was calculated with two-tailed Student′s t-test built in GraphPad Prism (San Diego, CA, USA).

## 3. Results

### 3.1. Construction and Characterization of BoHV-1 UL30-HA and UL42-FLAG Expression Plasmids

**Figure 1:**
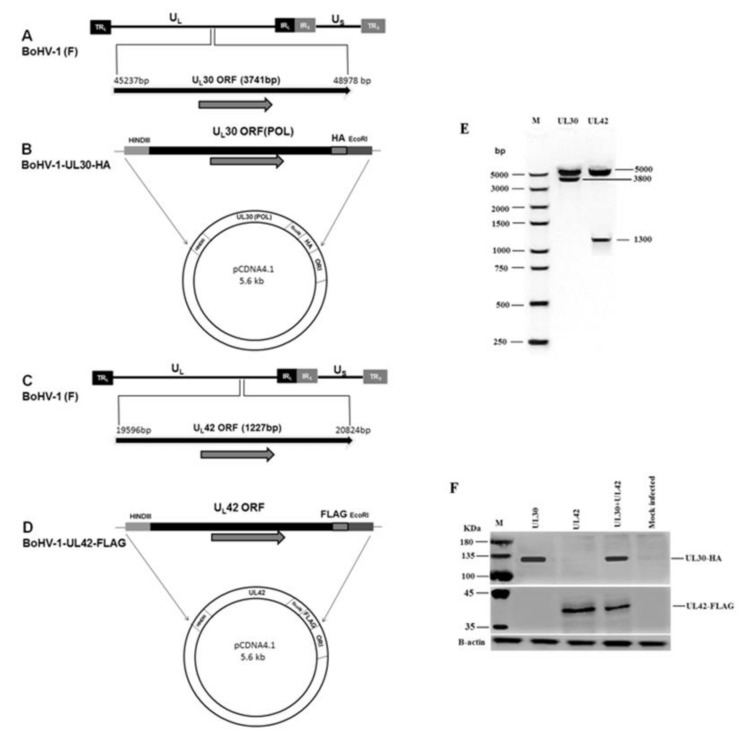
Construction and characterization of BoHV-1 UL30-HA and UL42-FLAG expression plasmids. (**A**) Schematic representation of the BoHV-1 genome (140 kb), along with unique short (US), unique long (UL), terminal repeat (TR), and internal repeat (IR) sequences. Position of UL30 (POL) is located between 45237 nt and 48978 nt on the BoHV-1 genome. (**B**) UL30 (POL) gene with restriction sites HindIII and EcoRI and the HA tag at C terminus was cloned into the vector pcDNA4. (**C**) Position of UL42 located between 19596 nt and 20824 nt on the BoHV-1genome. (**D**) UL42 containing restriction sites HindIII and EcoRI and the FLAG tag at C terminus were cloned into pcDNA4. (**E**) Restrictive digestion with Hind III and EcoRI. (**F**) SDS-PAGE/Western blotting assays were performed to detect the indicated fusion proteins. MDBK cells were transfected with indicated plasmids. At 24 h post transfection, the cells were processed for SDS-PAGE/Western blotting assays and the fusion proteins pUL30-HA and pUL42-FLAG were detected with monoclonal antibodies against either HA or FLAG tags. A representative blot of three independent experiments was shown.

To analyze the interaction between pUL30 and pUL42 in a cellular context, we generated recombinant expression plasmids encoding for both proteins, in a pcDNA4 backbone (Figure 1A, C). In order to allow simultaneous detection of both proteins, the UL30 and UL42 coding sequences were flanked at the 3′ end with the coding sequences for HA and FLAG tags, respectively, thus allowing the expression of C-terminally tagged proteins (Figure 1B, D). To verify correctness of the constructs, both plasmids were cut with specific restriction enzymes. The resulting reaction products were confirmed by agarose gel electrophoresis and the specific sizes of 3.8 kb for UL30 gene and 1.3 kb for UL42 gene (Figure 1E). By SDS-PAGE/Western blotting experiments, we could show that both plasmids encoded the expression of pUL30-HA (130 kDa) and pUL42-FLAG (40 kDa) fusion proteins in MDBK transfected cells (Figure 1F).

### 3.2. BoHV-1 UL30 and UL42 form a Complex in the Absence of other Viral Proteins

**Figure 2:**
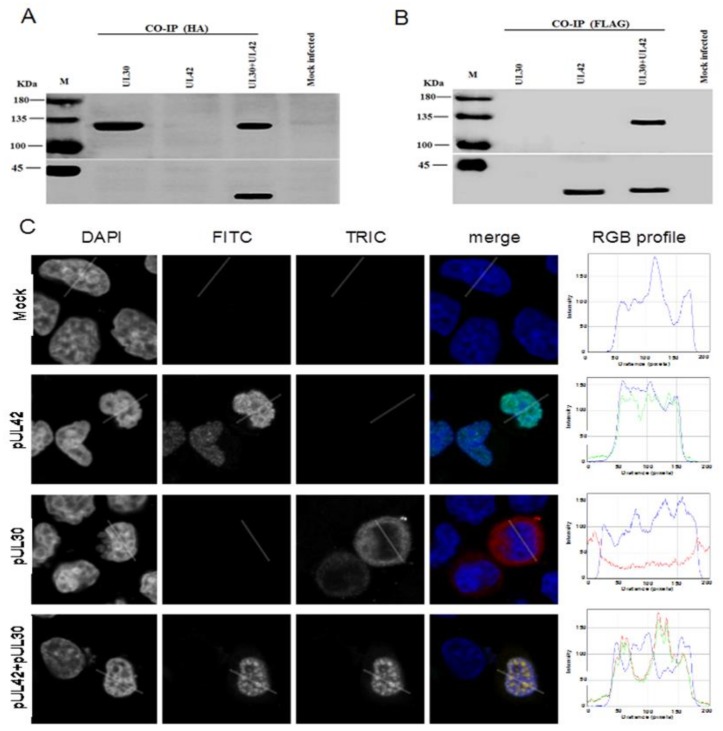
Expression of pUL42 is required for nuclear transport of pUL30 in the absence of other viral proteins. MDBK cells were transiently transfected to express the indicated fusion proteins and the cell lysates were subjected to Co-IPs by using anti-HA (**A**) and anti-FLAG (**B**) antibodies. Immunoprecipitated proteins were subjected to SDS-PAGE/Western blotting assays to detect the pUL30-HA and pUL42-FLAG fusion proteins using anti-HA (upper panels) or anti-FLAG (lower panels) monoclonal antibodies. The positions of the protein markers (M) are indicated on the left. Data shown are a representative one of three independent experiments. (**C**) MDBK cells were transiently transfected to express the indicated fusion proteins, processed for in situ immunofluorescence and subjected to CLSM analysis using anti-HA or anti-FLAG monoclonal antibodies. The cell nuclei (DAPI) along with the pUL42-FLAG (FITC) and the pUL30-HA (TRITC) fusion proteins are shown, in grayscale, along with respective RGB merged images of the three channels (merge). RGB profile plots relative to the indicated areas were shown on the right panels (RGB profile). Images are representative of three independent transfection experiments.

In order to investigate if pUL30 and pUL42 could form a complex in a cellular environment in the absence of other viral proteins, a CO-IP assay was performed. To this end, MDBK cells were transfected with the UL30-HA and UL42-FLAG expressing plasmids, either alone or in combination, and cell lysates were subjected to CO-IP using either anti-HA or anti-FLAG monoclonal antibodies. As expected, the anti-HA monoclonal antibody immunoprecipitated the pUL30-HA from the cellular lysates transfected with UL30-HA expressing plasmid alone or co-transfected with both UL30-HA and UL42-FLAG expressing plasmids (Figure 2A). Importantly, when UL42-FLAG was co-expressed with UL30-HA, it could also be co-immunoprecipitated by the HA monoclonal antibody. Similarly, the anti-FLAG monoclonal antibody immunoprecipitated the pUL42-FLAG from the cellular lysates transfected with UL42-FLAG expressing plasmid alone or co-transfected with both pUL30-HA and pUL42-FLAG expressing plasmids (Figure 2B). Importantly, when pUL30-HA was co-expressed with pUL42-FLAG, it could also be co-immunprecipitated by the FLAG monoclonal antibody. Thus, our findings demonstrate that pUL30 and pUL42 interact in the absence of other BoHV-1 proteins in a cellular context.

### 3.3. BoHV-1 pUL42 is Necessary and Sufficient for Nuclear Localization of pUL30

Since BoHV-1 replicates in the nucleus of an infected cell, the DNA polymerase holoenzyme (pUL30 and pUL42) has to move into the nucleus. We initially scanned their sequences for the presence of putative NLSs. Our analysis identified a cNLS at the C-terminus of pUL42 (GGA**RKR**P**R**AD-389), whereas no putative NLS could be identified on pUL30. We therefore decided to investigate the subcellular localization of pUL30-HA and pUL42-FLAG when they were transiently expressed alone or together in a cellular context, in the absence of other viral proteins. MDBK cells were transfected alone or together with these two plasmids, and the subcellular localization of pUL30-HA and pUL42-FLAG was analyzed by immunofluorescence/CLSM. Consistently with our bioinformatics analysis, when separately expressed, the pUL30-HA protein was predominantly localized in the cytoplasm, whereas pUL42-FLAG was strongly accumulated in the nucleus of transfected cells with a punctuate pattern (Figure 2C). Intriguingly, upon co-expression, both pUL30 and pUL42 accumulated in the nuclei of co-transfected MDBK cells, co-localizing in punctuate structures (Figure 2C). Our data suggest that pUL30 nuclear transport is dependent on pUL42, and therefore, that BoHV-1 DNA polymerase holoenzyme is translocated to the nucleus as a complex.

### 3.4. IVM Inhibits Nuclear Accumulation of BoHV-1 pUL42 Nuclear Accumulation

**Figure 3:**
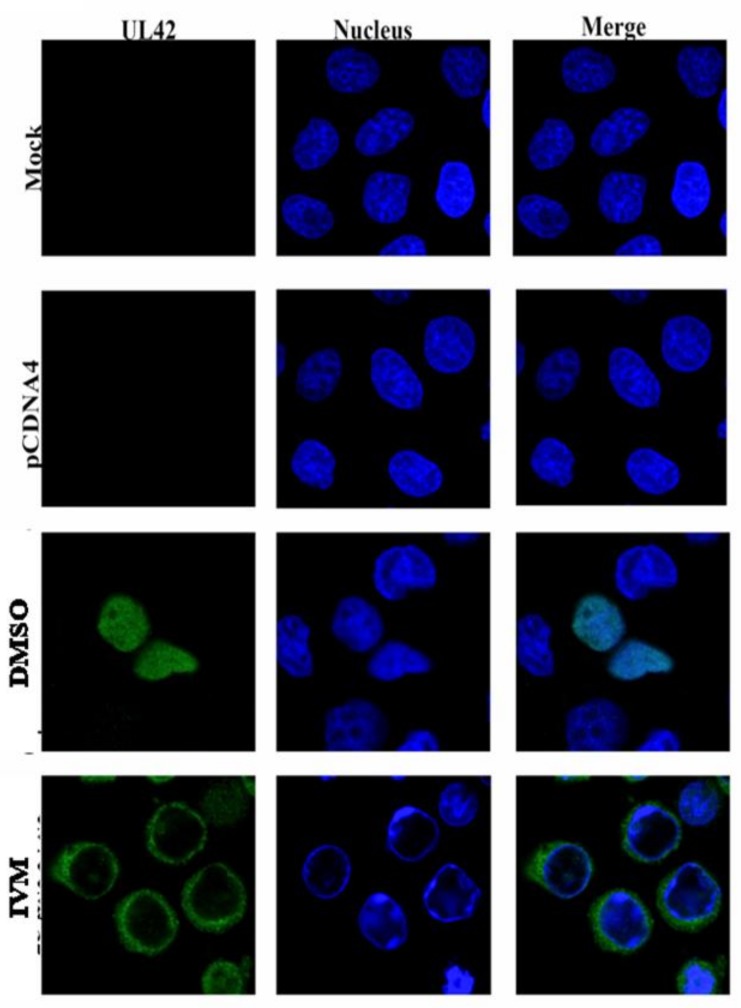
IVM impairs nuclear localization of BoHV-1 pUL42.MDBK cells were transiently transfected with pcDNA4-UL42-FLAG in the presence and absence of IVM (25 µM), processed for in situ immunofluorescence and subjected to CLSM analysis at ×63 magnification objective using an anti-FLAG monoclonal antibody. The pUL42 protein is shown in green (FITC); the nucleus is shown in blue (DAPI); and merged images of the two channels are shown on the right (merge).

Since IVM is a potent inhibitor of importin α/β mediated nuclear import, we hypothesized that it could interfere with nuclear accumulation of pUL42. MDBK cells were transfected with pcDNA4-UL42-FLAG plasmid in the presence or absence of IVM. At 18 h after transfection, the cells were fixed and processed for confocal microscopy examination. As shown in Figure 3, pUL42 was exclusively localized into the nuclei of transfected cells without drug treatment. However, treatment with IVM strongly reduced nuclear accumulation of pUL42 (Figure 3). These data suggest that pUL42 is transported to the nuclei via the Importin α/β pathway and that the process can be inhibited by IVM.

### 3.5. IVM Impairs BoHV-1 Life Cycle 

**Figure 4:**
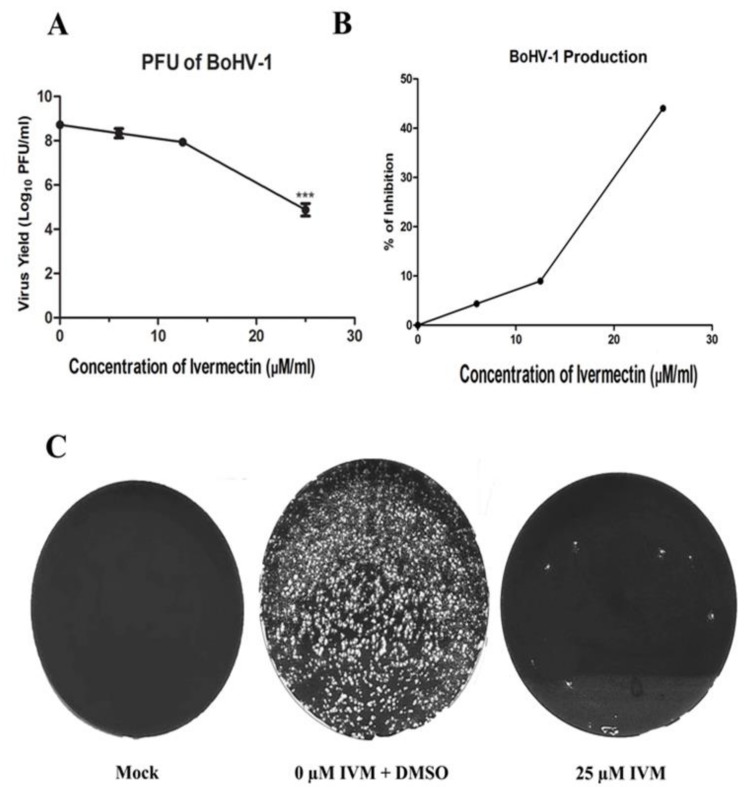
IVM impairs BoHV-1 replication. (**A**) MDBK cells were infected with BoHV-1 at MOI of 0.1 PFU/mL and subsequently treated with various concentrations of IVM and DMSO as control. At 24 h post infection, viruses were harvested through three cycles of freezing-thawing, and the virus titers were determined by plaque assay. Data shown are the mean ± SD from three independent experiments. Significance values were calculated using Student’s t test and determined by *p<0.05, **p<0.01, and ***p<0.001 relative to DMSO treated cells. (**B**) Percentage inhibition of BoHV-1 production after IVM treatment as compared to the control. (**C**) The results of plaque assay at 10^−4^ dilution of IVM treated and untreated cultures.

To test whether IVM has antiviral activity against BoHV-1, we performed virus replication assay. MDBK cells were infected with BoHV-1 at MOI of 0.1 PFU/cell and treated with increasing concentrations of IVM as indicated in Figure 4. At 48 h PI, the titers of progeny viruses were determined by PRAs. IVM inhibited BoHV-1 replication in a dose-dependent manner (Figure 4A). The virus titers were decreased by nearly four logs in the presence of 25 µM IVM (Figure 4C). There was ~44% inhibition in virion production from the infected cells treated with 25 µM IVM, as compared to non-treated infected cells (Figure 4B). Very similar results were obtained after infection at MOI of 1 PFU/cell, indicating that the observed reduction in BoHV-1 production observed in IVM-treated cells is not MOI dependent (Figure S1).

### 3.6. IVM does not Markedly Affect Cell Viability.

**Figure 5:**
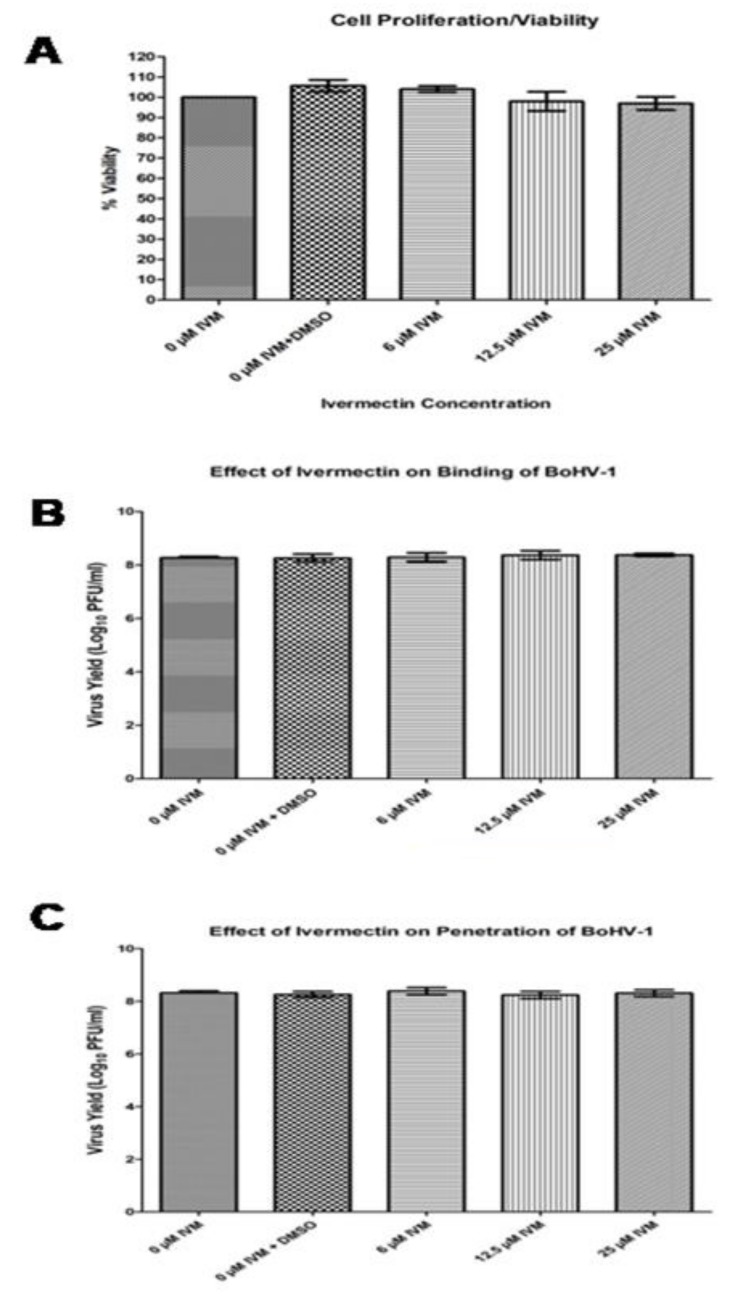
IVM does not affect cell viability, virus binding and internalization. (**A**) MDBK cells were incubated with increasing concentrations of Ivermenctin for 48 h. Cell viability was then measured using an MTT assay. Values represent the mean± SD of the three independent experiments. (**B**) BoHV-1 was pretreated with various concentrations of IVM at 37°C for 1 h and incubated with MDBK cells at a MOI of 0.1 PFU/cell at 4 °C for 1 h. The unbound viruses were removed by washing cells with PBS, and the cells were incubated at 37°C for a further 48 h. (**C**) MDBK cells were incubated with BoHV-1 at MOI of 0.1 PFU per cell at 4 °C for 1 h; the unbound viruses were removed by washing cells with PBS. Then the cells were treated with increasing concentrations of IVM, and incubated at 37 °C for 30 min. The cells were subsequently washed with citrate buffer (pH 3.0) to remove residual viruses and cultured in maintenance media at 37 °C for 48 hrs. The virus production in (B) and (C) was determined by PRAs. Data shown are the mean ±SD from three independent experiments. Statistical significance was calculated using Student’s t test according to *p<0.05, **p<0.01, and ***p<0.001.

We aimed at dissecting which stage of theBoHV-1 life cycle is blocked by IVM. To this end, we initially assessed its effect on cell viability by MTT cell proliferation assays. MDBK cells were treated with increasing concentrations of IVM, and the cell morphology was analyzed by light microscopy at 24 and 48 h post-treatment. No visible morphological changes were observed in cells treated with any of the tested IVM concentrations. The cytotoxicity of IVM was further assessed on MDBK cells using MTT. As shown in Figure 5A, MDBK proliferation rate was not affected significantly at 48 h post drug exposure. 

### 3.7. IVM Does Not Interfere With BoHV-1 Attachment and Entry. 

To test whether IVM affects BoHV-1 attachment to the cell membrane, we treated viruses with increasing concentrations of IVM at 37 °C for 1 h and then added the pretreated virus at MOI of 0.1 to MDBK cells at 4 °C for 1 h. Unbound viruses were washed out with PBS, and then maintenance medium was added, followed with incubation at 37 °C for 48 h and measurement of virus production by PRAs. Our results indicate that IVM treatment did not cause any significant change in virus titers as compared to cells untreated or treated with solvent only, suggesting that IVM did not affect BoHV-1 binding to cell surface.

We similarly tested whether IVM affects BoHV-1 penetration by performing a synchronous infection. Viruses were initially incubated with MDBK cells at a MOI of 0.1 PFU/cell for 1h at 4°C to allow virus attachment but not viral entry. Subsequently, unbound viruses were removed, various concentrations of IVM were added, and the temperature was shifted to 37°C for 30 min to allow viral entry. Subsequently, drugs and residual viruses were removed, cells were incubated in maintenance media at 37°C for a further 48 h, and the virus production was determined by PRAs. We found no significant difference in virus production among the untreated cells, cells treated with solvent only and cells treated with various concentrations of IVM (Figure 5C), indicating that IVM treatment did not generate detrimental effects on BoHV-1 penetration into host cells.

## 4. Discussion

The pathobiology of BoHV-1 is similar to that of human herpes simplex virus 1 (HSV-1); both viruses establish lifelong latent infection after a short replication cycle [6,31]. Therefore, BoHV-1 can serve as an animal model of human herpesvirus infection and anti-BoHV-1 strategies could help control the herpesvirus infection not only in animals but also in humans. In addition, since the currently available BoHV-1 vaccines provide suboptimal protection against the disease [32], it is necessary to develop a novel antiviral strategy for the infected animals [33].

Herpesviruse DNA replication occurs in the nuclei of infected cells and is mediated by a set of viral proteins, including a DNA polymerase holoenzyme composed of a catalytic subunit and a PAP, conferring processivity to the holoenzyme [34]. Intriguingly, herpesviruses have been reported to use two different strategies to ensure nuclear localization of their DNA polymerase holoenzyme. Indeed, while all *Herpesviridae* PAPs studied so far have been shown to localize to the nucleus when expressed in the absence of other viral proteins due to the presence of a functional cNLS in their C-terminus, the same is not true for their DNA polymerase catalytic subunits [10]. Indeed, while DNA polymerase catalytic subunits from HSV-1 and HCMV can translocate to the nucleus autonomously, those from KHSV, EBV, HHV-7, and PRV can be imported to the nucleus only when complexed with their PAP to form the complete holoenzyme [10]. Our data suggest that BoHV-1 evolved similarly to the latter members, because nuclear import of the catalytic subunit pUL30 appears to be dependent on piggy-backing from its PAP pUL42, which possesses a cNLS in its C-terminus (Figure 2). Indeed, only co-expression of pUL30 with pUL42 allowed nuclear localization of pUL30.

IVM was recognized as a ‘Wonder Drug’ due to its great beneficial effects on human and animal health. It was firstly discovered in 1970s by Satoshi Omura and William Cecil Campbell [35]. Thereafter, it has been broadly used as an antiparasitic medicine in both humans and animals. For example, IVM has been used against gastrointestinal and respiratory nematodes and arthropods in wild and domesticated animals [36]. Recently, the discovery that IVM is a potent inhibitor of importin α/β-dependent nuclear transport [37] broadened its potential application to the control of a number of viral infections ranging from DENV and ZIKA to HIV-1 [22,37,38,39]. Our results demonstrated that IVM inhibits pUL42 nuclear import (Figure 3), thereby impeding BoHV-1 viral replication (Figure 4) but without causing cell toxicity and affecting virion binding and internalization (Figure 5), and strongly suggest that inhibiting nuclear transport of DNA polymerases for Herpesviridae members could be a viable antiviral strategy, as shown in the case of PRV infection [22]. There was almost 4 log reduction in virus titer after using IVM; however, the virus titer was reduced 2.47 logs after using Acyclovir, an antiviral drug of herpes viruses [40]. Our findings thus expand the antiviral repertoire of IVM. Due to current limited control options against BoHV-1 infection, use of IVM against anti BoHV-1 infection could be a potential therapeutic option in future. Current work in our laboratories is focused on testing the ability of IVM to inhibit BoHV-1 replication in vivo and its efficacy in preventing the occurrence of clinical signs and transmission. 

## Supplementary Materials

The following are available online at https://www.mdpi.com/2076-2607/8/3/409/s1, Figure S1: Antiviral activity of Ivermectin against BoHV-1.
