# Supplementary material for: Ivermectin Inhibits Bovine Herpesvirus 1 DNA Polymerase Nuclear Import and Interferes With Viral Replication
Source: Microorganisms. 2020 Mar 13;8(3):409. doi: 10.3390/microorganisms8030409 (PMC7143239; doi:10.3390/microorganisms8030409)

Figure S1. Antiviral activity of Ivermectin against BoHV-1. MDBK cells were treated with indicated concentrations of Ivermectin following viral infection at MOI of 1 (A) and 0.1 (B).. Viral titers were determined using plaque assay. The results shown represent the means ±SD (error bars) from three independent experiments. Statistical significance was calculated using Student's *t* test according to *$p < 0.05$, **$p < 0.01$, and *** $p < 0.001$.

Supplementary Figure 1

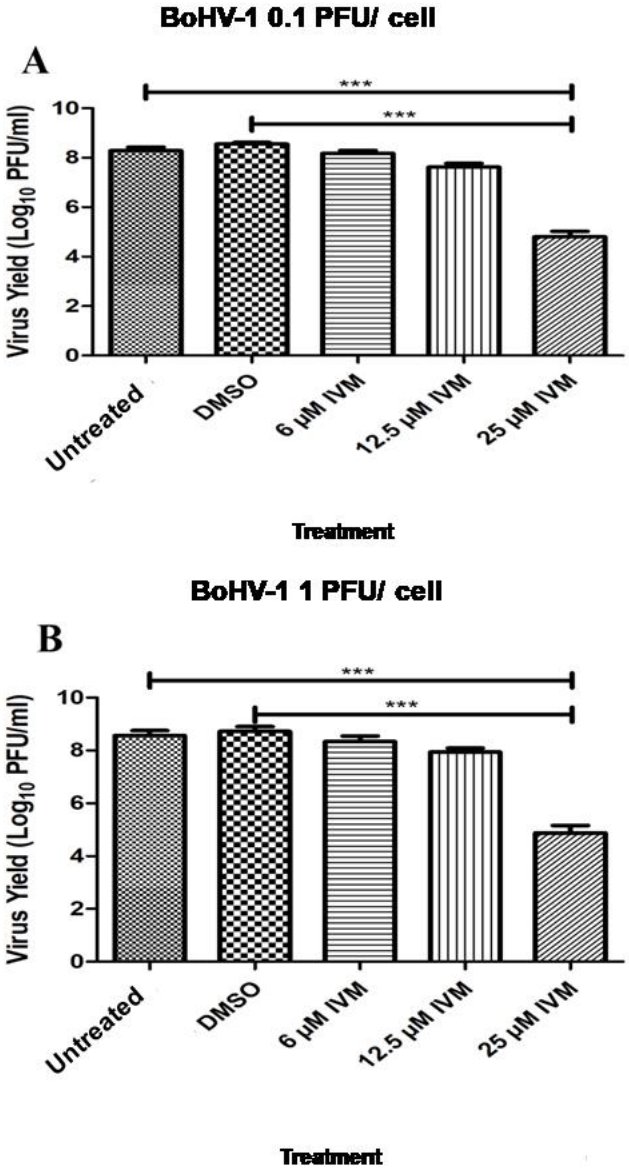

Supplement: Supplementary file 1 [file microorganisms-08-00409-s001.zip › Supplementary Figure S1.pdf]
